# Examining the Effectiveness of Automated Acoustic Recording Units for Recording Predator‐Related Disturbances in Colony Nesting Birds: A Case Study

**DOI:** 10.1002/ece3.72379

**Published:** 2025-10-21

**Authors:** Dilan Praat, Gregory Schmaltz

**Affiliations:** ^1^ University of the Fraser Valley Abbotsford British Columbia Canada

**Keywords:** automated recording units, bioacoustics, disturbances, remote, species interactions

## Abstract

As habitat destruction and human expansion pushes wildlife to ever shrinking habitats, new methods are needed to monitor and assess the impacts of disturbances on ecosystems. Automated recording units (ARUs) may provide a cost effective and minimally invasive way of monitoring disturbances and behavioral responses under these changing conditions. ARUs are gaining prominence in avian research, replacing in‐person observers in various surveys and in tracking the movement of individual birds. Researchers have investigated the reliability of ARUs in these studies, but investigations into their reliability in detecting behavioral events are lacking. The main objective of this case study is to investigate if disturbance data from predation events in Ardea herodias fannini (heron) colonies can adequately be obtained from bioacoustics recordings and used in research to substitute for in‐person observations. ARUs were placed at two heron colonies and accompanied by in‐person observers. Both sources recorded minor or major predatory disturbances to the colonies with minor disturbances being a single heron responding and major disturbances being multiple herons responding. The records of detected disturbances from each observation method were compared. We found that ARUs were able to distinguish major disturbances from other calls. There was no considerable difference between major disturbances detected by ARUs or by in‐person observers. However, the ARUs did have marginally less success when trying to detect minor disturbances. This was attributed to ARUs providing purely auditory cues as opposed to some visual cues that human observers occasionally rely on. When monitoring remote colony nesters with distinct auditory calls, ARUs can provide a cost effective and scalable substitute for in‐person observers. These data can be easily repurposed for other research questions, stored for long‐term studies to find gradual changes in behavior, or used to study unexpected or rapid changes to an environmental variable.

## Graphical abstract

Automated recording units (ARUs) have soared in popularity within the field of avian research and have been used in various studies to replace human observers when collecting data. There is, however, a lack of research into the actual reliability of ARUs when compared to in‐person observers, especially when investigating avian behavior. This case study investigated this by examining how ARUs and in‐person observers compare when detecting predatory disturbances in colony nesting birds and found that ARUs can substitute for in‐person observers.

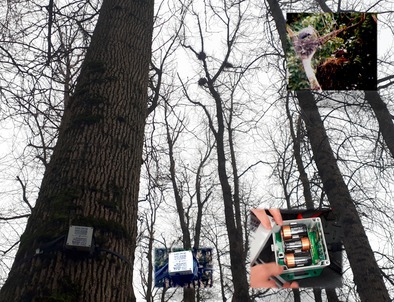

## 1 Introduction

With the relatively recent development of passive forms of acoustic monitoring, researchers can collect data on wildlife without the need for direct human observations (Teixeira et al. 2019). Continued advancements have led to relatively cost‐effective automated acoustic recording units (ARUs) (Darras et al. 2019; Potamitis et al. 2014; Sugai et al. 2019). These programmable units record acoustic data in the field while unattended for extended periods of time (Brandes 2008; Teixeira et al. 2019). Data collected can easily be stored after the initial project and then recalled for future studies as new research questions emerge (Teixeira et al. 2019). ARUs can be used to study any vocalizations with minimal impacts on the subject species' habitat or behavior (Garland et al. 2020; Sugai et al. 2019; Teixeira et al. 2019). The widespread adoption of ARU‐based research methods has led to many applications in fields such as bird and bat biology, marine science, and citizen science (Armstrong et al. 2021; Brandes 2008; Sousa‐Lima et al. 2013; Sugai et al. 2019). Researchers interested in monitoring avian populations are no longer restricted to costly and time‐consuming traditional field methods such as mist netting, point counts, or in‐person surveys (Brandes 2008; Shonfield and Bayne 2017). These advances present an opportunity to gather large datasets and, in turn, could open new avenues in avian behavioral research (Sugai et al. 2019; Teixeira et al. 2019).

Researchers have used ARUs to determine the location of individuals during point counts and to track habitat occupancy (Alquezar and Machado 2015; Chronister et al. 2025; Wood et al. 2020). ARUs have also been used to investigate the presence of sensitive, rare, or nocturnal species in marshes, wetlands, or mountainous habitats (Bobay et al. 2018; Goyette et al. 2011; Pérez‐Granados et al. 2018; Sidie‐Slettedahl et al. 2015). Additional studies have used ARUs to investigate the activity levels of rare nesting seabirds on offshore islands (Buxton et al. 2013). In addition, researchers were able to observe seabird nocturnal vocalizations to understand the scope and impacts of invasive or eradicated rat species (Buxton et al. 2013). While ARUs have been used primarily to estimate broad species presence, recent studies have begun to investigate behavioral patterns (Celis‐Murillo et al. 2009; Darras et al. 2019; Pérez‐Granados et al. 2019; Sierro et al. 2023). This includes research on song selection in songbirds during courtship and on vocalization timings in response to the frequency overlap of calls with similar pitch or songs (Chronister et al. 2023; Sierro et al. 2023). ARUs have also been employed to investigate the shifting calling activity of two species of tinamou ( *Crypturellus undulatus* and *Ortalis canicollis* ) in response to differing climate conditions (Pérez‐Granados and Schuchmann 2021). Additional studies have used large‐scale ARU arrays to understand habitat occupancy across environmental gradients such as forest coverage at different life stages in Great horned owls *Bubo virginianus* or to investigate the breeding habitat selection of Canada warblers *Cardellina canadensis* (Chronister et al. 2025; Czarnecki et al. 2024).

Careful consideration must be given to the expanding behavioral applications of passive acoustic monitoring (PAM) and ARU‐based approaches. For instance, there is a need to ascertain the degree to which observations drawn from ARU data correspond to human observations. Investigations into the reliability of ARUs in point counts have demonstrated that, depending on the conditions, ARUs can be a useful tool with comparable results to an in‐person observer when measuring species diversity, especially when ARUs and in‐person observations are done in conjunction (Digby et al. 2013; Drake et al. 2021). Further research aimed to understand the detection ranges of different models of ARUs when compared to in‐person observers utilizing the playback of vocalization of specific species (Yip et al. 2017). These studies demonstrated that while human observers can have a greater detection range and that environmental variables such as foliage density can hinder ARU performance, ARUs still provide reliable and useful data when measuring species richness (Alquezar and Machado 2015; Winiarska et al. 2024; Yip et al. 2017). However, to our knowledge, there has been little research into the correspondence of ARU data and human observations when evaluating or distinguishing discrete avian behavioral vocalization events. It is this gap that this case study aims at investigating.

The main objective of this case study is to investigate if recordings taken from ARUs can substitute for in‐person observations when collecting behavioral data during predation disturbances in Pacific Great Blue Heron *Ardea herodias fannini* (referred to henceforth as herons) colonies. Herons were selected as our model species for several reasons. First, herons have a documented and observable vocalized response (distress call) to predation attempts which is distinct from other heron vocalizations (Vennesland 2002). Second, once threatened, multiple members of a heron colony will often engage in the distress call simultaneously, with a full colony's response being heard by our observers up to 600 m away. When an event such as attempted predation caused this vocalized distress response from a heron or herons, we would define that as a disturbance. Third, herons form relatively compact, isolated, and long‐term colonies in tall trees ranging from 2 to 200 nests, making their nesting locations easy to predict and observe by both in‐person observers and remote acoustic equipment while also eliminating the risk of other colonies appearing on acoustic recordings (COSEWIC 2008). Fourth, B.C. herons have adopted associative nesting behaviors with their primary predator, Bald eagles *Haliaeetus leucocephalus* (henceforth referred to as eagles), forming predator–protection relationships (Jones et al. 2013). Eagles maintain a territory of roughly 200 m that they aggressively protect from other eagles, and herons have begun to exploit this by nesting within that territory (Jones et al. 2013). While still being preyed upon by the “resident eagle pair”, herons nesting within the territorial influence of the eagles have a higher nesting success rate due to an overall reduction in predation by roaming eagles (Jones et al. 2013). The resident eagles fly past or at the heron colony during predation attempts throughout the nesting season, creating a reliable source of predation disturbances. Finally, these disturbances can be localized to a single nest, or they can be on a larger scale and disturb multiple nests, indicating a stressful event for large portions of the colony with potentially non‐consumptive effects (Cresswell 2008; Vos et al. 1985). These differences will allow us to investigate not only the reliability of ARU spectrograms in recording predatory disturbance events, but also to assess how observers using ARU spectrograms rate predatory disturbances of differing severities. These disturbances can be classified as major disturbances where multiple herons are threatened, with the response being loud, and minor disturbances which are created from single, individually threatened herons. Minor disturbances tend to be more subdued both in terms of intensity and duration and hence, should be harder to detect. We aim to investigate whether observers analyzing ARU data and in‐person observers can distinguish these two types of distress calls from each other and from other types of stress‐related vocalizations (e.g., intraspecies disputes over resources, nesting locations, or space within the colony). We predict that the two observation methods of in‐person observation and ARU manual spectrogram analysis will result in similar rates of distress calls caused by disturbances to the colony (i.e., picking only calls that resulted from predatory attempts rather than disputes between herons) (prediction one). Second, we predict that both methods will agree on when these disturbances occurred (prediction two). Finally, we predict that both methods will be more reliable when detecting major disturbances as opposed to minor disturbances (prediction three).

## 2 Methods

### 2.1 Location Selection

**FIGURE 1:**
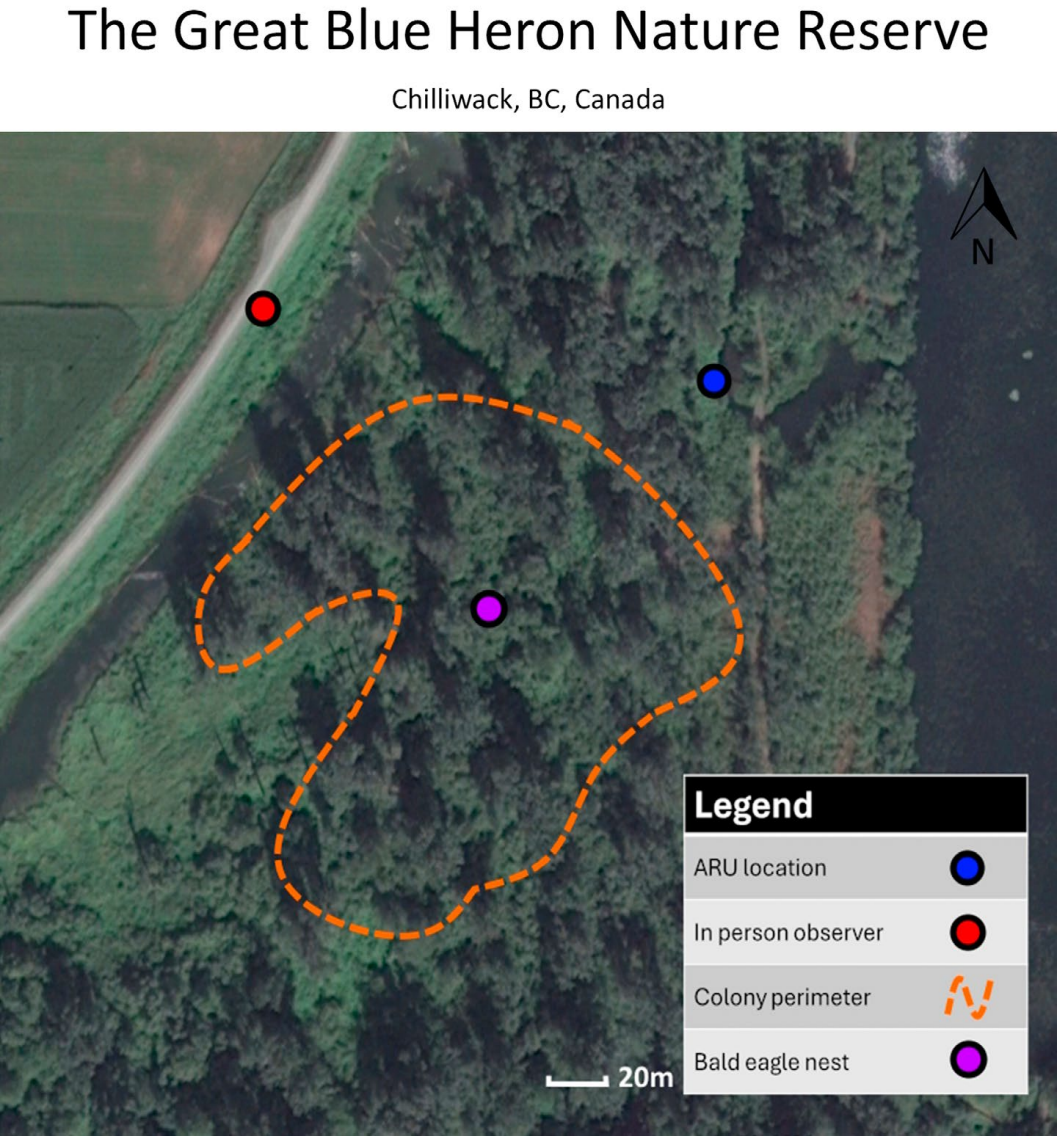
The layout for the GBHNR colony research site. The distance and location of the ARU and in‐person observer from the colony perimeter. Heron nests ranged in height from 15 to 30 m.

**FIGURE 2:**
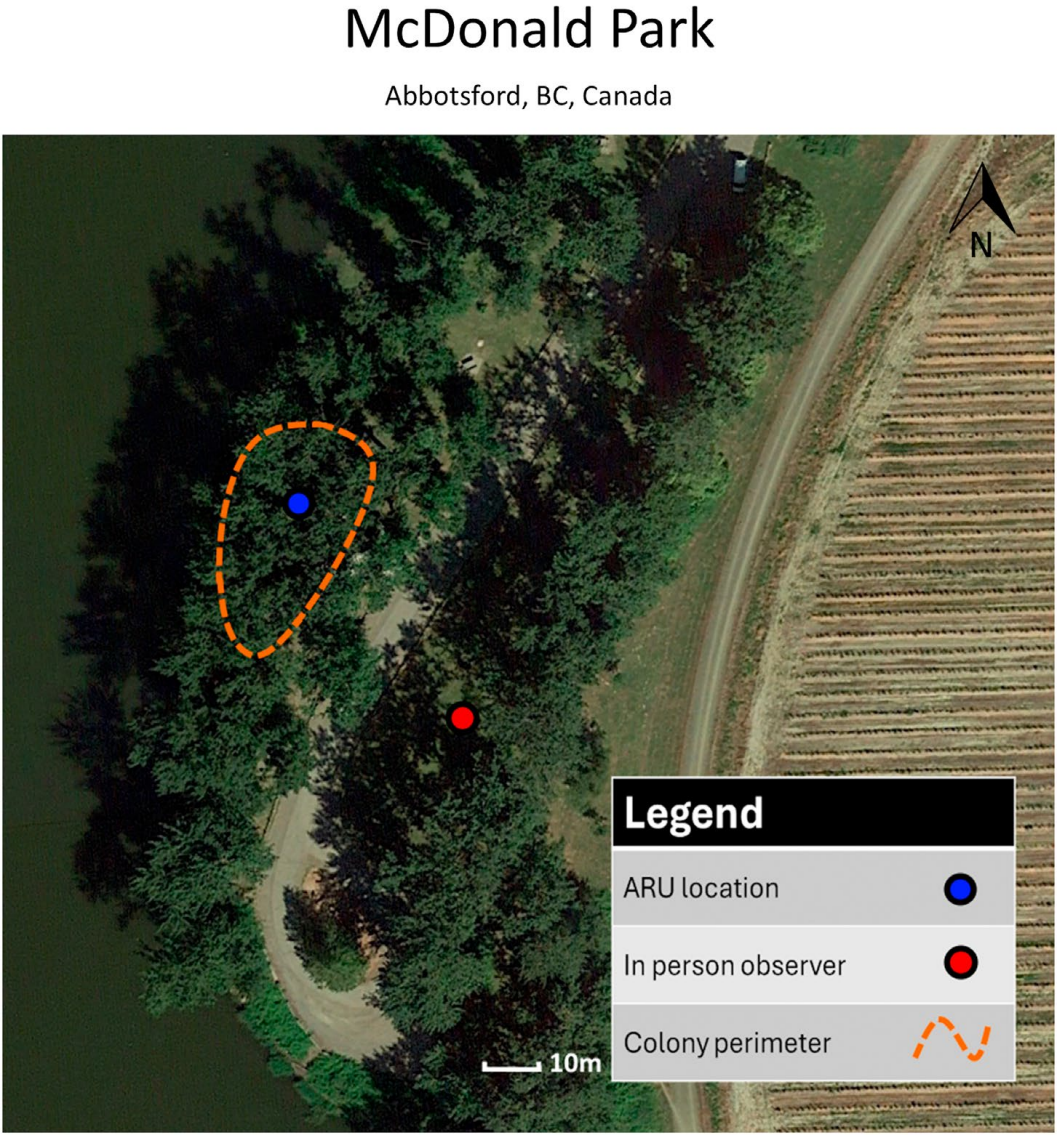
The layout of the MDP colony research site. Note the nearby human activity and lack of an eagle nest. Heron nests ranged in height from 15 to 25 m.

We selected two active heron colonies during the 2020 nesting season (February through June). The first colony was located at the Great Blue Heron Nature Reserve (referred to as GBHNR) (Chilliwack, BC, Canada, 49.093147, −122.049435), which contained 72 heron nests and one eagle nest within 100 m of the colony (Figure 1). The second was at Macdonald Park (referred to as MDP) (Abbotsford, BC, Canada, 49.089039, −122.138147), which contained 11 heron nests and no eagle nest at the colony site (Figure 2). The closest eagle nest to the MDP heron colony was over 500 m away, which was well beyond the 200 m protective territorial influence of an eagle's nest (Jones et al. 2013). To examine the role of ARU recordings in detecting disturbances, these two colonies were selected specifically for their differing setups regarding the presence of eagle nests. We expected the predator–protection model to provide an adequate number of disturbance events to test the method at GBHNR. However, some studies have shown that these disturbances can be rare, and the nature of the predator–protection model meant there was the chance that an unguarded colony (MDP) could be preyed upon (disturbed) more frequently by roving eagles (e.g., non‐resident eagles) (Jones et al. 2013; Van Damme and Colonel 2007). Since there was no way of knowing with certainty which colony would be disturbed more frequently prior to the study, we set up observations at both GBHNR and MDP. Given the scope of our case study and the large number of hours spent in the field at each site, these two selected colonies should allow us to draw meaningful conclusions on the efficacy of ARUs in both scenarios. The ARUs used in this study were two Swift terrestrial passive ARUs (Cornell Center for Conservation Bioacoustics, Cornell University, Ithaca, NY, United States). The ARU at GBHNR colony was 1.5 m off the ground in a small tree and was outside the colony perimeter (Figure 1). The ARU at the MDP colony was placed 3 m up in a tree directly below the center of the colony, with the lowest nests being 15 m away (Figure 2). The GBHNR colony was in a secluded area of a park closed off to the public and showed signs of sensitivity to the presence of humans. As such, we placed the ARU outside the perimeter of the colony. We placed the ARU at the MDP colony directly below the colony as the herons were habituated to the presence of humans at this recreational park. The placements of ARUs and visual in‐person observers were selected to maximize the efficacy of each respective method given the limitations of each location (e.g., colony sensitivity to humans or visual obstruction). Locating both observation methods at the same observation point at either colony would have had a negative impact on either of the methods (decreased visibility from foliage if too close or a low signal‐to‐noise ratio for ARUs if too far). Despite the variance in ARU placement for our case study, initial observations showed that disturbances were clearly recorded on both devices.

### 2.2 ARU and In‐Person Observations

The ARUs were programmed to record continuously for 24 h a day, 7 days a week with a sample rate of 16 kHz and a microphone analogue gain of 33 dB using the Swift Configuration Tool (Cornell Center for Conservation Bioacoustics, Cornell University, Ithaca, NY, United States). This 24/7 recording schedule was selected with future long‐term studies in mind, with the plan of investigating dawn, dusk, or nocturnal disturbances. The sampling rate was chosen as a compromise between sound quality and prolonged battery life to minimize maintenance visits to the colonies. These units were deployed in late February 2020, with SD cards (128 GB) and batteries being extracted/refreshed every 28–30 days throughout the breeding season. The colonies were observed for 20 h a week by up to four in‐person observers during the breeding season. These observations were split into randomized observation periods ranging from 0.75 h to 6 h during daylight hours ranging from 6:00 am to 6:00 pm where continuous monitoring for heron disturbances was conducted. Each observer underwent 6 h of training in the field and a further 6 h in the lab with audio files to ensure consistency in the data collected, with 80% of the data being recorded by one observer. When a disturbance was observed, details were taken including the date, time, duration, the severity of the colony's response, and the source of the disturbance (if discernible). We classified a disturbance to the colony as any event (typically the presence of a predator) that caused the herons to respond with their distress call and threat display for 3 s or more. The severity of the disturbances was categorized as either a minor disturbance or a major disturbance, with a single heron disturbed being classified as a minor disturbance, and more than one heron disturbed being a major response. Disturbances were classified as separate disturbances if there was a 30 s gap with no heron distress calls being detected or observed. At both locations, the in‐person observer station was on top of a 5 m tall river dike with good visibility on at least 70% of the nests throughout the breeding season. The in‐person observer station at the GBHNR colony was 50 m from the nearest nest and 200 m from the farthest nest (Figure 1). The in‐person observer station was 50 m away from the nearest nest at the MDP colony (Figure 2).

### 2.3 Spectrograms

**FIGURE 3:**
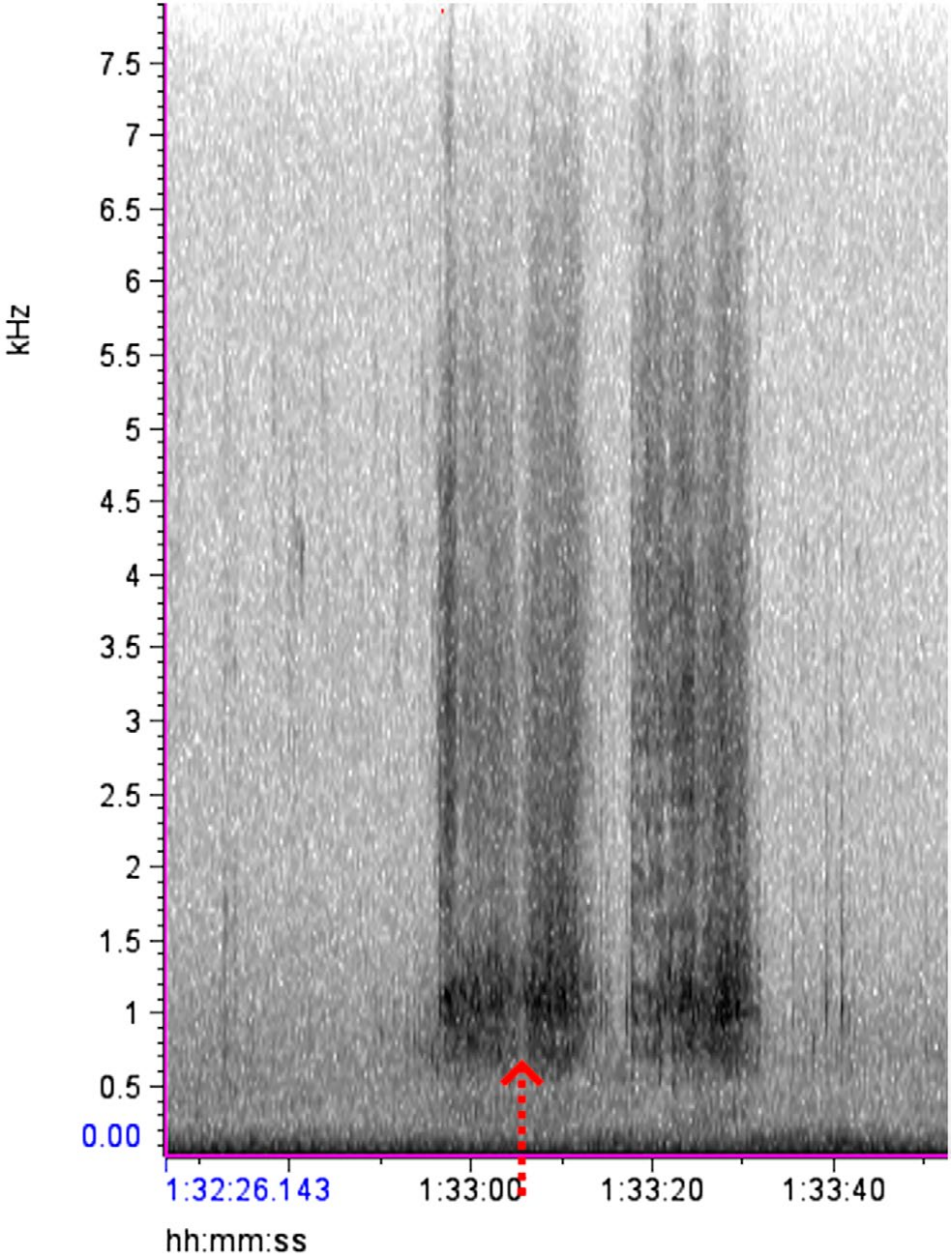
Spectrogram from Raven Lite 2.0, showing a major heron disturbance. The red arrow shows that the gap does not cut cleanly through the enter spectrogram, indicating multiple herons are responding to the threat.

**FIGURE 4:**
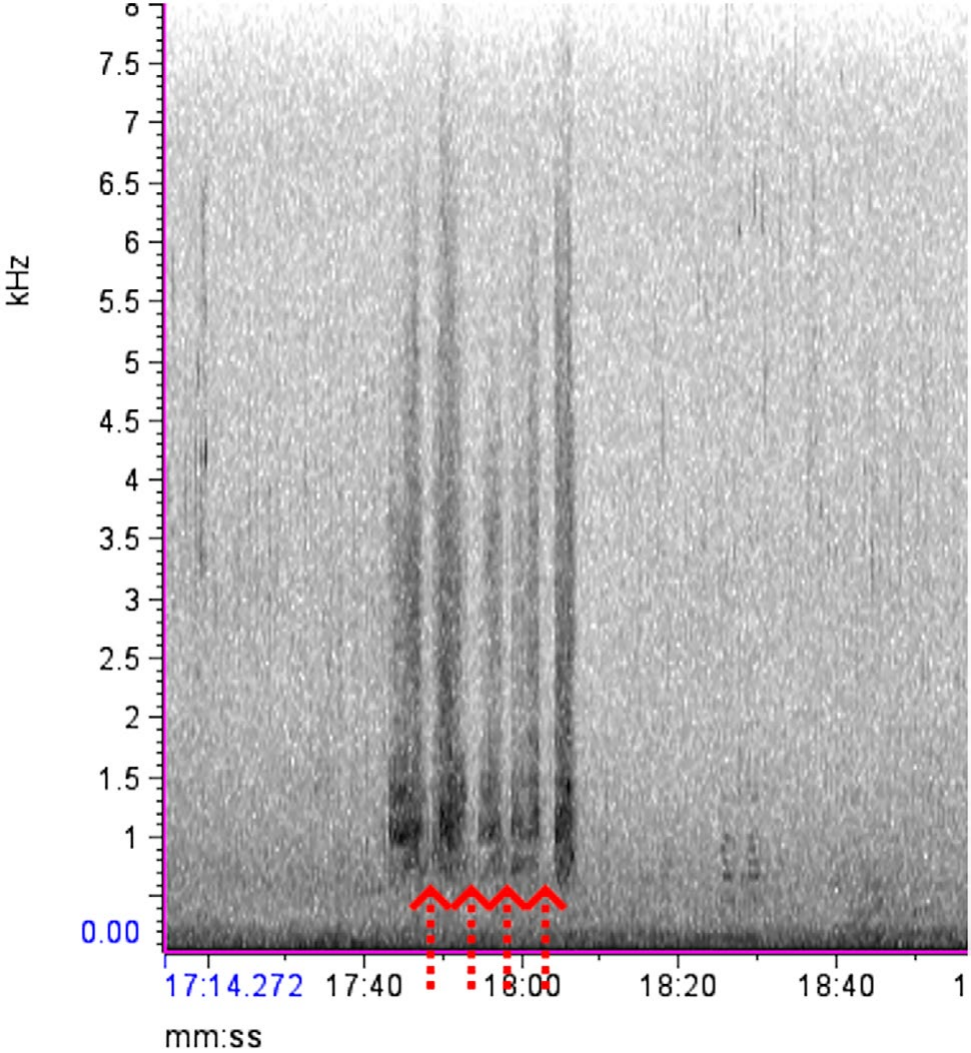
An example of a single minor disturbance, from 17:42 (min:s) to 18:08. The bars are clearly separated (red arrows), meaning a single heron is making the call as it must take a breath after each distress call.

**FIGURE 5:**
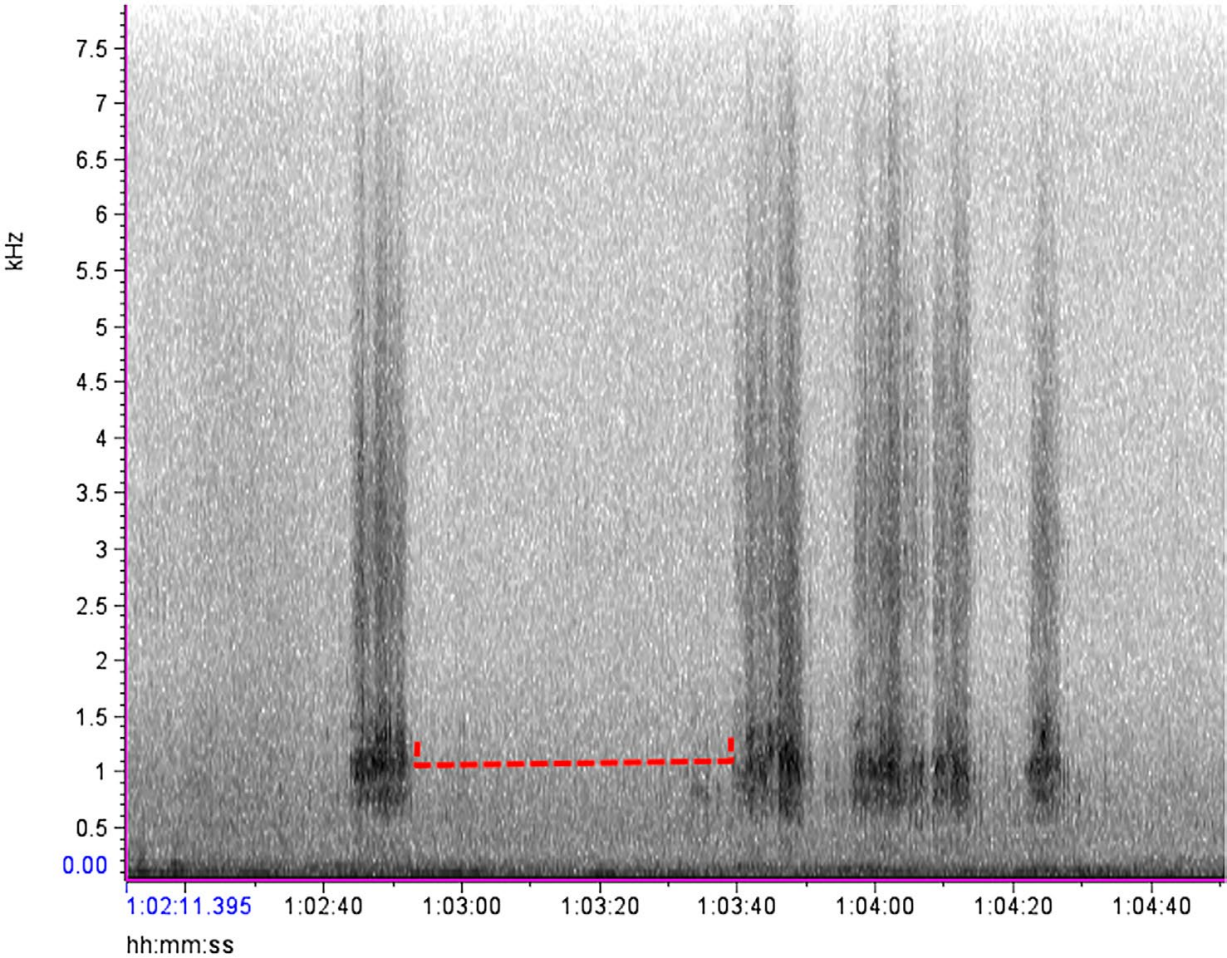
An example of two major disturbances. The first one is from 1:02:43 (h:min:s) to 1:02:52, and the second is from 1:03:41 to 1:04:28. The disturbances are separated by more than 30 s (dashed red line), resulting in two separate disturbances. Both have periods where individual calls cannot clearly be distinguished, making them both major disturbances.

**FIGURE 6:**
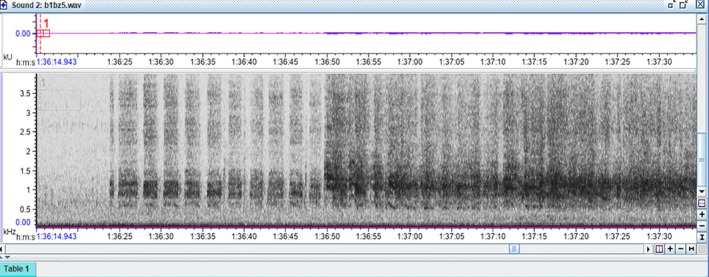
From 1:36:20 (h:min:s) to 1:36:50, only one heron was responding to a disturbance. After 1:36:50, more herons responded, and because there was no 30 s break of silence, this entire disturbance was treated as a single major disturbance. This was not uncommon, as an isolated nest on the periphery of the colony may detect an incoming predator and respond before the predator moves closer to the center of the colony.

Using our preliminary observations and the description of the distress calls by Vennesland (2002), various audio samples of heron distress calls made during predation attempts were isolated for reference. As predicted, these vocalizations were found to be distinct from other heron vocalizations, starting off with groaning croaks, followed by a series of loud squawks or screeches with short gaps that were repeated until the threat was removed (Vennesland 2002). The spectrograms of this behavior formed vertical bars starting at 0.5 kHz with the densest part of the spectrogram showing at 0.8–2 kHz, and the upper limit of the call extending up past 4 kHz (Figure 3). Once the ARU files and observer data sets were extracted from the field, the dates and file names were encrypted for a double‐blind analysis so that the investigator (D. P.) analyzing the audio files was unaware of the date, time, or location the recordings came from. The encrypted ARU data was displayed via spectrograms in the program Raven Lite 2.0 (Cornell Center for Conservation Bioacoustics, Cornell University, Ithaca, NY, United States). Each 2 h wav file was manually scrolled for evidence of heron distress calls. After finding a disturbance on the spectrogram, the audio file was played to confirm the distress call, and then the disturbance event was classified as minor or major by looking for gaps between the spectrogram marks or by listening for the number of herons manually. If the marks were separate and clearly spaced out, it was classified as a minor disturbance as only a single bird replied (Figure 4). If the bars appeared faded together, overlapped, or were overly long, they were classified as a major disturbance as that would require multiple herons to respond to cover the gaps between each individual's calls (Figure 5). This also means that if a disturbance started as a minor disturbance but then developed into a major disturbance, the entire episode was treated as a single major disturbance (Figure 6). After analyzing the ARU data, the file names were decrypted and the audio files were matched to the in‐person observations for comparison.

### 2.4 Statistics and Data Analysis

The data were evaluated in two ways using the Krippendorff's alpha test to assess the inter‐reliability between the two observation methods (Hayes and Krippendorff 2007). We used SPSS Statistics (v29). We then loaded the kalpha.sps macro developed by Hayes and Krippendorff (2007) to assess the agreement between the observation methods in how they rate events by assigning an alpha (*α*) derived from the test. Krippendorff's alpha can range from 1 to −1, with an *α* = 1.0 being complete agreement, *α* = −1.0 being complete disagreement, and anything above an *α* = 0.8 being considered excellent agreement when making conclusions about the data (Hayes and Krippendorff 2007; Marzi et al. 2024). We first took the number of disturbances recorded over an observation period and divided them by the length of the period (in hours) to obtain a rate of disturbances per hour. Converting the number of disturbances per period to rates of disturbances per hour allowed observation periods to be compared directly. In this case study, we are reporting standard deviations along with means for observation periods and rates of disturbances. One of the goals of this case study was to compare whether observations drawn from ARU spectrograms give similar results to in‐person visual observers (prediction one). Therefore, our first test was used to investigate whether the results of each detection method provided similar rates of disturbances; not whether they were detecting the same specific disturbances (i.e., prediction two). For instance, in an observation period, if the in‐person observer detects a major disturbance at 10:13 am and the observer using spectrograms detects a major disturbance at 10:55 am, both methods would have a rate of 1 disturbance per hour, while not in agreement on when that disturbance occurred. This was important since predation events can be infrequent or rare occurrences (Van Damme and Colonel 2007). If both methods agreed that there were 0 disturbances in an observation period, they would both be providing similar observations. These rates per observation period were compared using Krippendorff's alpha with the corresponding rate of the other observation method. For instance, major disturbances per hour via in‐person observation were compared to major disturbances per hour via ARU observation. Our second test was to investigate whether both methods agreed on the existence and rating of individual disturbance events (prediction two). We isolated each individual disturbance to investigate whether the observation methods actually agreed on the presence and/or severity of each individual disturbance event at a given time. The case‐by‐case agreement via Krippendorff's alpha was assessed twice. The first assessment used all three ratings including major disturbance, minor disturbance, and no disturbance. The second assessment used only two ratings, major disturbance and no disturbance, with minor disturbances being downgraded to no disturbances. Since major disturbances have a wider impact on more herons, they are also more likely to have more meaningful biological non‐consumptive effects. Minor disturbances were added to test the robustness of these methods of monitoring (prediction three).

## 3 Results

We conducted fieldwork over 53 observation periods for a total of 166 h, with an average duration of 3.1 h (±1.82 h). We recorded for 73 h at MDP and 93 h at the GBHNR. Manual verification of the spectrograms showed that a total number of 81 disturbances were detected by reviewing ARU recordings, 48 of which were major disturbances, while the in‐person observers recorded a total of 86 disturbances, 50 of which were major disturbances (Table 1). We calculated four rates for each observation period, including major disturbances per hour and all disturbances per hour for each of the two observation methods (ARU spectrogram analysis or in‐person observation) (Table 1). For our first test, we found that there was strong agreement between both categories of measurements when comparing rates (*α* = 0.957 for all disturbance rates and *α* = 0.995 for major rates only). Since all disturbances recorded during the study occurred at one location (GBHNR), we repeated the test for the observation periods at the GBHNR location only. There was still excellent agreement (*α* = 0.940 for all disturbance rates and *α* = 0.993 for rates of major disturbance only).

**TABLE 1:** Observed total number of disturbances detected by in‐person observers and ARUs.

	In‐person	ARU
Total disturbances	86	81
Major disturbances	50	48
Minor disturbances	36	33
Mean of rates (all disturbances per hour ±SD)	0.59 (±1.03)	0.55 (±1.02)
Mean of rates (major disturbances per hour ±SD)	0.37 (±0.76)	0.36 (±0.76)

On a case‐by‐case basis, we found that when sorting between the three disturbance ratings, there was very strong agreement (*α* = 0.935) between observation methods and even stronger agreement when rating disturbances as either major disturbances or no disturbances (with minor disturbances being treated as no disturbance) (*α* = 0.953). In total, there were five cases out of 86 where the in‐person observer detected a minor disturbance that the observer analyzing ARU data missed entirely (5.81% of cases). There were two further cases where the in‐person observer detected a major disturbance while the observer analyzing ARU recordings identified the disturbance as a minor disturbance, creating a disagreement. In the first discrepancy, the in‐person observer noted that two herons vocalized a distress call in sync. This spectrogram showed bars with clear gaps as if a single heron called, paused to inhale, then called again. The second discrepancy occurred when the in‐person observer noted an eagle predation attempt on a heron 100 m outside the colony. This initiated a response from a total of two herons, the heron outside the colony that the eagle attempted to prey upon, and another heron at the furthest edge of the colony from the ARU.

**FIGURE 7:**
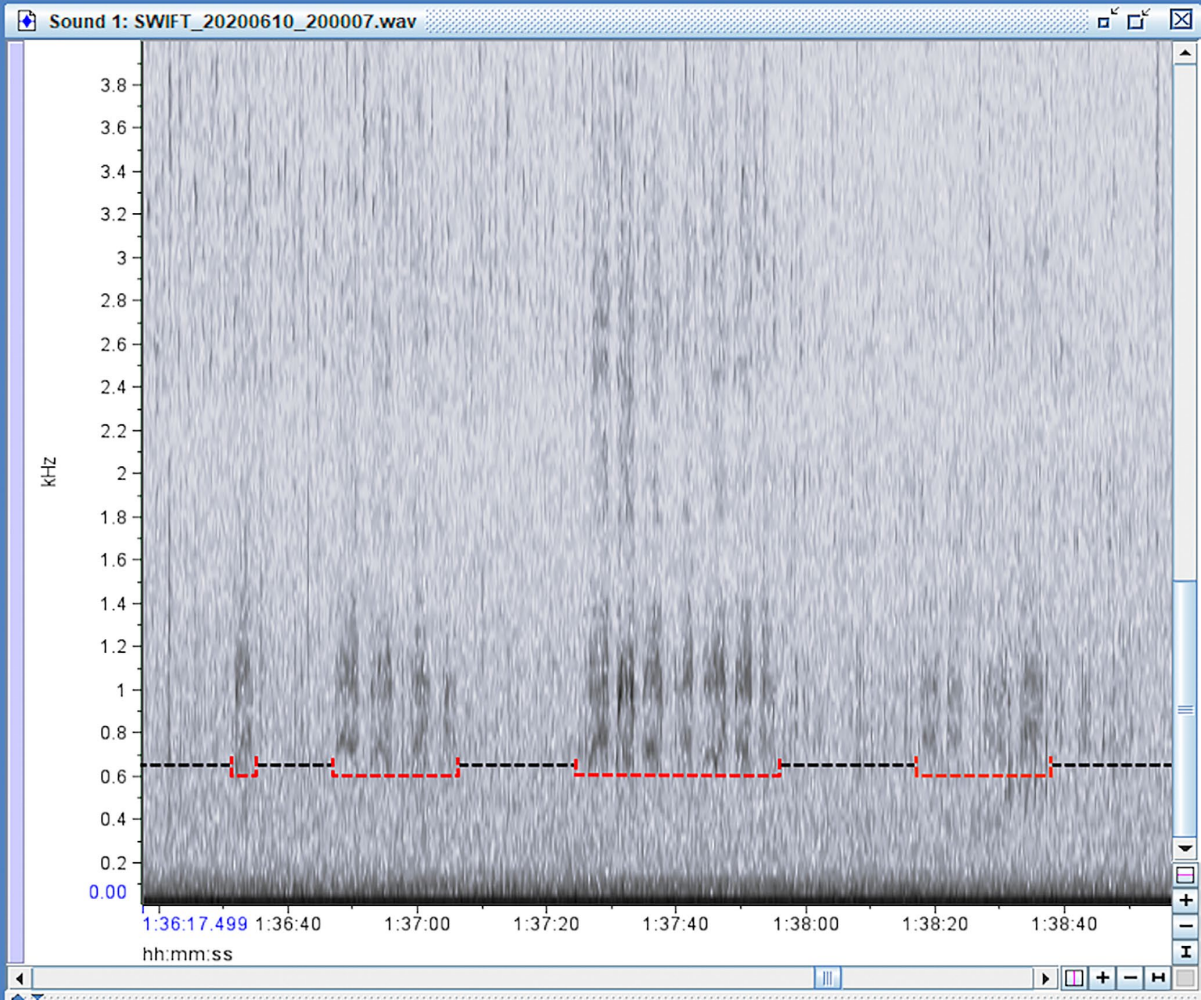
Spectrogram generated by a heron's distress call in response to a raccoon attempting to climb into a heron's nest, confirmed by the in‐person observer. The raccoon would attempt to get into the nest (red sections), then retreat a few meters to rest (black sections), before trying again.

In general, disturbances were typically brief, most ranging from 5‐30 s in length while on occasion a predator would position itself near a heron nest, which would greatly prolong that disturbance. When using in‐person observations to determine the cause of disturbances, we found that eagle‐related disturbances were the most frequent source of disturbance in a colony, making up 79 (91.9%) of 86 disturbances detected (Table 2). Of these eagle‐related disturbances, 54 (62.8%) were eagles passing through or near the colony (within 30–50 m) and 25 (29.0%) were eagles engaging in predation attempts (e.g., diving, abrupt turns, entering nests) on herons or heron nests. A further three (3.5%) disturbances were caused by attempts of a raccoon *Procyon lotor* to prey on a heron nest (Table 2). The raccoon tried to climb up a tree and into a heron nest on three occasions during broad daylight, all within the same observation period. This resulted in a series of individual distress calls from the single heron defending its nest, with the calls appearing similar on the ARU spectrogram to those triggered by an eagle pass or predation attempt. These raccoon‐triggered disturbances were roughly 2–4 min long, during which the raccoon would relent and climb 1–2 m down the branch below the nest for 10–25 s then make another attempt to enter the heron nest. Afterwards, the raccoon descended a few meters down the tree to rest for 1–2 min, then the raccoon would make a new series of attempts to get into the heron nest. This resulted in a single heron making an intermittent series of distress vocalizations for up to 2–4 min followed by a 1–2 min pause as opposed to the regular 5–30 s isolated disturbances with constant vocalization caused by eagles. The result on the spectrogram was three separate minor disturbances (Figure 7). When compared to other minor disturbances, the ARU spectrogram generated by the raccoon‐related predation attempts decreased in intensity as they continued. This differed from most eagle‐related disturbances, which were shorter and consistent in intensity. For the purposes of our study, we treated each of these predation events as a single minor disturbance (three total) as our project focuses on the detection methods of predation disturbances. A further four minor disturbances (4.65%) showed all the characteristics of a disturbance (distress call, threat display, etc.) but the source of these disturbances could not be determined by either the in‐person observer or the spectrograms.

**TABLE 2:** Causes of disturbances as recorded by the on‐location in‐person observer.

Source of disturbance	Disturbances caused	% of All disturbances	Major disturbance caused	% of Major disturbances
Eagle‐related	79	91.9%	50	100.0%
Eagle‐related (Predation attempt)	25	29.1%	24	48.0%
Eagle‐related (Pass)	54	62.8%	26	52.0%
Other predator	3	3.5%	0	0.0%
Unknown	4	4.6%	0	0.0%

Of the 50 major disturbances reported by the in‐person observer, all were related to eagles. In 26 (52.0%) of the major disturbances, we observed that an eagle was passing through or near the colony without appearing to engage in predation attempts. The remaining 24 (48.0%) major disturbances were all direct predation attempts, with an eagle diving at nests or herons, landing in heron nests, or attacking perched adults or fledgling herons. The in‐person observer noted that there was only one predation attempt that did not trigger a major response; this was an eagle preying upon a heron outside the colony, and this caused only a single heron within the colony to respond. In 48 of the 50 major disturbances, the observer analyzing ARU spectrograms agreed with the severity of the disturbance. In the two disturbances that the observer analyzing ARU spectrograms categorized as minor disturbances while the in‐person categorized as major disturbances, one was an eagle predation attempt, and the other was a pass. Of all disturbances, 26 (30.2%) were followed up by a subsequent disturbance within 10 min, 13 of which were major disturbances being followed by another major disturbance (Table 3). Furthermore, there were a total of six cases where a disturbance would begin as an established minor disturbance lasting 3 s or longer that would then escalate to a major disturbance.

**TABLE 3:** A breakdown of paired sequential disturbances that occurred within 10 min of each other.

Sequence of disturbances	Number of paired events	% of all disturbances
Major disturbance followed by minor disturbance	4	4.7%
Minor disturbance followed by major disturbance	5	5.9%
Minor disturbance followed by minor disturbance	4	4.7%
Major disturbance followed by major disturbance	13	15.1%

## 4 Discussion

### 4.1 Findings

Our results suggest that ARUs can be used to investigate behaviors relating to predatory disturbances. There was excellent agreement between the in‐person observer and the observer analyzing ARU recordings in detected disturbances, both when considering overall rates (prediction one) and on a case‐by‐case basis of individual disturbances (prediction two). We found strong support that both observation methods detected similar rates of disturbance (*α* = 0.957 for all disturbance rates and *α* = 0.995 for major disturbance rates only), suggesting that in terms of disturbance, ARUs can substitute for in‐person observers (prediction one). This also suggests that observers analyzing ARU spectrograms can differentiate between calls made by herons in response to predatory threats as opposed to calls arising from other behaviors or events within the colony. The lowest alpha of *α* = 0.935 came from the case‐by‐case basis when investigating all disturbances (3 categories of disturbance). This suggests that while there is still strong/excellent agreement (prediction two), using ARU recordings to observe minor disturbances leads to marginally less reliability when compared to major disturbances only (*α* = 0.953). This supports our third prediction. As minor disturbances only have a single heron actively calling, these events could be considerably quieter. If these minor disturbances were on the opposite side of the colony from the ARU, they could have a poor signal‐to‐noise ratio and thus be much harder to detect. Since a single heron is affected during these minor events, the biological impact of these disturbances is likely minimal when compared to major disturbances, where well over 100 herons could be stressed (Vennesland 2002). These minor disturbances may also be more subject to the directionality of the call, as a call projected upward at an aerial predator may not show as reliably on a recording unit stationed below and outside the perimeter of the colony. This means the farthest distress calls could have originated up to 200 m away from the ARU and may again have suffered from a poor signal‐to‐noise ratio. Since placement height, surroundings, and even wind direction can alter ARU performance, this could impact ARU‐related research and should be considered in future studies (Thomas et al. 2020). Factors such as wind and rain, which often vary from hour to hour, can also have a negative effect on ARU performance (Turgeon et al. 2017). This variation could be compounded by the lack of consistent and frequent checks of local weather at the actual location of the ARU unit, which was not possible at the GBHNR due to the sensitive nature of the herons at that colony. While general weather data was collected by the in‐person observer at the beginning of every observation period, the distance between the GBHNR ARU (in low to midstory foliage close to the colony) and the in‐person observation post (on a 5 m high, open dike) was roughly 100 m. This means that wind measurements at the in‐person observation site would likely not be reflective of the situation at the location of the ARU. Further research also showed that ARUs can suffer from microphone degradation when in the field for extended periods of time (Turgeon et al. 2017). This can have a negative impact on the ARU's microphone sensitivity, especially at lower decibels (Turgeon et al. 2017). The ARUs used in this study were only 2 years old by the end of the research period but had spent roughly 240 days recording in the field. Since this damage could affect ARU microphone sensitivity at lower decibels, this could also have an impact on the ARU's ability to record minor disturbances, as the heron's minor distress calls are at lower decibels than major ones. Therefore, the possible microphone degradation compounded with the issues outlined earlier may have resulted in readings on the spectrogram that were too faint, likely explaining why some minor disturbances were not detected on the spectrograms. These shortcomings could be compensated for in future research by placing additional ARUs around the colony perimeter. Researchers could also select an ARU that could record throughout the breeding season without maintenance and set it up at the center of the colony prior to the arrival of the herons. While neither of those approaches were used for our study, either method would have reduced the distance between the vocalizing herons and the ARUs down to 100 m or less. Alternatively, due to the ARUs' ability to allow the observer analyzing the spectrograms to repeat and review every segment of data, the observer analyzing ARU spectrograms may have been able to define, reclassify, or reject minor disturbances more accurately from other calls than the in‐person observer. Since the in‐person observer was recording disturbances via notetaking, there was limited time for the observer to record the details of sporadic (and often rapid) periods of disturbances. Most importantly, with regards to our research, there were no cases where the observer analyzing ARU recordings detected a disturbance when the in‐person observer detected nothing. There were two major disturbances observed by the in‐person observer that the observer using ARU spectrograms assessed as minor disturbances. The first was a major disturbance with two herons calling in sync, briefly responding to an eagle flying 50 m from the far edge of the colony, and the second was a heron preyed upon by an eagle 100 m outside the colony with one nest responding at the furthest end of the colony (thus two herons making distress vocalizations, one of which was outside the colony limits). This suggests that when compared to the in‐person observer, observers using ARU data are more likely to underrate the severity of a disturbance. This may be due to the ARU data being more susceptible to environmental conditions, resulting in a false negative detection (i.e., the ARU not detecting a disturbance).

To investigate further the reliability of ARU recordings as a stand‐in for an in‐person observer, we made additional comparisons. Rates of disturbance per hour took into consideration the 33 observation periods (93 h) during which there were no disturbances, with the comparisons having almost perfect agreement for both all disturbances (*α* = 0.957) and major disturbances only (*α* = 0.995). In our 166 h of in‐person observations, we observed that eagles caused 79 disturbances (0.476 disturbances per hour) and eagles made 25 predation attempts (0.151 predation attempts per hour). These results are higher than those reported in other studies, suggesting that there is a wide range in the number of naturally occurring disturbances. Norman et al. (1989) observed 0.097 incursions per hour and 0.046 disturbances per hour. Vennesland (2002) observed 0.246 eagle incursions per hour. Jones et al. (2013) reported 0.06 eagle incursions per hour and a predation attempt rate of 0.03 attempts per hour. This variation suggests that predation varies with colony location, year, and surrounding habitat. ARUs as an observation method of disturbances could assist in gathering enough data to get a more conclusive understanding of eagle and heron nesting dynamics. Many of the other studies that investigated disturbances and rates of disturbance also had relatively short observation periods, and thus a limited time to monitor for disturbances as they traded off more observations at fewer colonies in favor of more colonies with shorter observation periods (Jones et al. 2013; Vennesland 2002). ARUs have the advantage of monitoring colonies continuously throughout the breeding period (nest building, incubation, fledging, etc.). As another good example of the wide range of naturally occurring disturbances, there were no disturbances detected at the MDP colony by either observation method. The MDP colony had a very high fledgling success rate that year with an average of more than 3.0 fledglings per nest in 2020. In comparison, other studies have reported 0.82 fledglings per breeding attempt (Vennesland 2002) or 0.9–1.0 fledglings per nest in other colonies not utilizing the territorial protection of eagles reported by Jones et al. (2013). This is more in line with typical breeding success of herons such as the 1.2 fledglings per nest at GBHNR in this study. The agreement in the lack of disturbances at the MDP colony in ARU data and in‐person observations suggests that both observers are able to differentiate disturbances from other calls, despite the wide range of vocalizations herons can produce from events such as older chicks fighting for food or an adult heron landing at the incorrect nest. The ARU spectrogram manual verification did not mistake any of these alternate vocalizations for disturbances.

### 4.2 Flaws and Drawbacks

One of the notable drawbacks of the observations made from ARU spectrograms is that since ARUs only collect acoustic data, it can be hard to interpret behavioral vocalizations pertaining to unexpected situations beyond the scope of the initial study. Furthermore, while the observer analyzing the ARU spectrograms noted that the distress calls provoked by the raccoon looked and sounded marginally different in that they were less intense and intermitted with frequent breaks, they were not able to say conclusively that it was caused by a raccoon or any other species. By extension, the in‐person observer reported that 92% of disturbances were related to eagles. The observer using ARU data could often detect the calls of eagles around the times of disturbance but was never able to state with certainty what was disturbing the colony. A disturbance event with similar auditory characteristics to the raccoon disturbance was also observed on the ARU spectrograms during the night in April 2020 at the GBHNR colony. This nocturnal disturbance lasted for nearly 2 h, and while it matches the raccoon disturbance spectrogram in appearance and character, we have no way to verify the actual cause. While a raccoon may be the probable cause as they have been known to prey upon heron nests at night, there are other nocturnal predators that may also prey upon heron nests (COSEWIC 2008). These types of observations highlight the importance of preliminary research into the characteristics and behaviors of the subject species to ensure that behavioral observations drawn from ARU recordings alone will lead to desirable and usable data.

In this case study, we manually analyzed the spectrograms. It took roughly 1–2 h for the spectrogram observer to analyze an average of 24 h worth of spectrograms. While this was practical in terms of two colonies for our study, multiplying the data set by more colonies would have quickly proven overwhelming in the time required to process data if such resources are limited and the goal is to analyze every single hour. However, an observer manually reading the ARU spectrograms was still much more efficient in the time required to observe disturbances than an in‐person observer. The development of artificial intelligence (AI) programs such as BirdNET or other similar programs to analyze bulk acoustic data automatically has essentially removed the need for manual ARU observations of the spectrograms, making this approach even more attractive (Chronister et al. 2025; Kahl et al. 2021; Stowell et al. 2019; Szymański et al. 2021). Further issues encountered with the ARU recordings were often related to human activities such as construction, quarry blasting, or the use of vehicles such as airplanes, helicopters, or automobiles. The sounds created by these activities and machines would often obscure the spectrograms, making them impossible to read for brief periods, especially when looking at lower frequencies.

### 4.3 Pros and Future Applications

Despite these possible limitations, there is enormous potential for this technique in behavioral research, with this case study demonstrating that ARUs can be used as a stand‐in for human observers, especially if used to detect distinct major disturbances in colony nesting birds. Major disturbances often lead to loud responses from the target species, leading to a high consistency in ARU detection rate. The ability to detect the more subtle minor disturbances with a high degree of confidence suggests that this technology can be applied to a wide range of behavioral responses, even in cases where vocalizations are more difficult to detect in terms of intensity or distinguishability from other vocalizations. In addition, we found it easier to classify disturbances using ARU spectrograms as opposed to in‐person observations. This was in part because there were clear definitions and boundaries between the two disturbance types on the spectrograms, with the ability to play back the disturbance if observers were unsure. Other advantages of ARUs observed in this study include the ability to exploit PAM to investigate unpredictable events. In 2019, the resident eagle nest at the GBHNR colony was lost in a storm partway through the colony's breeding season. ARUs placed were able to record nesting disturbances before and after the loss of the eagle nest, which may allow for future research to investigate how predatory pressure on the colony changed through the breeding season (Jones et al. 2013). Similarly, consistent PAM from ARUs could be used in behavioral research to monitor how species respond to unexpected human activity, how they respond to changes in habitat, or to compare changes in behavior and breeding success over multiple seasons (Buxton et al. 2013; Sugai et al. 2019; Teixeira et al. 2019). The use of ARUs can be especially useful when studying rare events such as in this study, as study subjects can be monitored 24/7, an impossible task for field workers.

In the context of behavioral research, ARUs could also be used in conjunction with other observation techniques such as in‐person observation, video observation, or other emerging technologies. For instance, drones have been used with thermal cameras to map heron colonies, and microphone arrays have been used to track the movements of individuals of a particular species (Rhinehart et al. 2020; Schedl et al. 2020). Researchers could either use these additional observation techniques to plan the positioning of ARUs for optimal data collection or use ARUs to scout different situations that researchers are interested in before committing additional resources to the location. One example of this application may be the future deployment of ARUs to sensitive areas to detect poachers. If poaching is detected or suspected, the ARU could be analyzed, and more expensive, specialized equipment could be directed to the appropriate areas. Research is already ongoing in the use of ARUs and AI to detect gunshots in jungle ecosystems (Katsis et al. 2022).

## 5 Conclusions

In this case study, we have provided evidence that ARUs can be a viable and reliable substitution for in‐person observations in behavioral research. In herons, when used to detect and rate the severity of predator disturbance events, the ARUs were shown to be an effective observation system. ARUs were also able to distinguish heron distress calls derived from predation‐related events from other behavioral calls. Limitations such as poor weather (e.g., windy conditions) or unexpected events (e.g., novel predators) highlight the usefulness of additional means of data collection, especially when setting up a new study system. For instance, in‐person observations or assistance from other forms of monitoring such as remote cameras or microphone arrays can provide additional information or context during sound data interpretation. After this initial work is completed, ARUs can be a force multiplier in the collection of audio‐based behavioral data, with the technology being especially effective when observing the group responses of a target species. Future studies will expand upon topics related to this case study. First, we will continue cataloging disturbances by including other factors such as additional colonies and looking into nocturnal disturbances. Second, we will evaluate changes in heron nest site selection in relation to eagle nest relocations. Third, we will assess whether we can deduce the cause of disturbances from ARU spectrograms by looking at vocalizations surrounding the disturbance (e.g., eagle calls). Finally, given the developments in the automation of the analysis of ARU data, future research will look at the reliability of AI software to detect disturbances as opposed to using manual scoring.

## Author Contributions

**Dilan Praat:** conceptualization (equal), data curation (lead), formal analysis (lead), investigation (equal), methodology (equal), project administration (supporting), writing – original draft (lead), writing – review and editing (supporting). **Gregory Schmaltz:** conceptualization (equal), data curation (supporting), formal analysis (supporting), funding acquisition (lead), investigation (equal), methodology (equal), project administration (lead), supervision (lead), writing – original draft (supporting), writing – review and editing (lead).

## Disclosure

Statement on inclusion: Our study aimed to investigate methods of monitoring local, at‐risk species in the Fraser Valley, Canada. Efforts were made to discuss research avenues with local non‐profit organizations and local experts and to gain a deeper understanding of the challenges facing species in urbanizing environments. Various students and professors at the University of the Fraser Valley were asked to engage with and learn about the topics and species being researched. Despite this, we feel that more could have been done to include the viewpoints of Indigenous peoples, and we will aim to seek their input and support in future research.

## Conflicts of Interest

The authors declare no conflicts of interest.

## Supporting information


**Data S1:** ece372379‐sup‐0001‐DataS1.xlsx.

**Data S2:** ece372379‐sup‐0002‐DataS2.xlsx.

## Data Availability

All the required data is uploaded as Supporting Information.
